# Association between culture of patient safety and burnout in pediatric hospitals

**DOI:** 10.1371/journal.pone.0218756

**Published:** 2019-06-24

**Authors:** Cintia de Lima Garcia, Italla Maria Pinheiro Bezerra, José Lucas Souza Ramos, Joseane Elza Tonussi Mendes Rossette do Valle, Maryldes Lucena Bezerra de Oliveira, Luiz Carlos de Abreu

**Affiliations:** 1 Setor de Pós-graduação, Pesquisa e Inovação, Centro Universitário Saúde ABC, FMABC, Santo André, SP, Brazil; 2 Faculdade de Medicina Estácio de Juazeiro do Norte, FMJ, Juazeiro do Norte, Ceará, Brazil; 3 Programa de Pós-graduação em Políticas Públicas e Desenvolvimento Local, Escola Superior de Ciências da Santa Casa de Misericórdia de Vitória, EMESCAM, Vitória, ES, Brazil; 4 Laboratório de Escrita Científica, Escola Superior de Ciências da Santa Casa de Misericórdia de Vitória, EMESCAM, Vitória, ES, Brazil; 5 Centro Universitário Doutor Leão Sampaio, Juazeiro do Norte, Ceará, Brazil; Nord University, NORWAY

## Abstract

**Introduction:**

Providing safety culture is the first and undoubtedly most important feature that patient care should have, as it is the basis for other measures. There are associations between Burnout Syndrome and lower perceptions of safety culture and greater risks in patient safety.

**Objective:**

To analyze the relationship between patient safety culture and burnout in pediatric hospitals.

**Method:**

This is a cross-sectional study with a quantitative approach performed with health professionals who work in pediatric hospitals located in the Metropolitan Region of Cariri, Northeastern Brazil. The study enrolled 148 professionals who performed direct health care for the child in three different hospitals. For the evaluation of the Patient Safety Culture, the version, translated and validated for Brazil, of the Survey on Patient Safety Culture (HSOPSC) questionnaire was applied and to evaluate the occurrence of Burnout, we chose the Maslach Burnout Inventory (MBI).

**Results:**

Among the dimensions of burnout that presented the most moderate to high, depersonalization and low professional achievement stand out. When considering the syndrome with the professional who presented a "high" score in only one of the three dimensions, it was identified that 44.6% presented the disease. All patient safety dimensions studied correlated with some dimension of burnout.

**Conclusion:**

The study evidenced the influence of all patient safety domains for the development of burnout syndrome in pediatric professionals. Also, it was identified that the organizational climate is the main determinant of burnout, especially in what refers to "teamwork between units".

## Introduction

"Patient Safety" is based on health sciences and day-to-day professional practice in the various work environments, offering the means to provide care without generating negative consequences for individuals under the care of the multiprofessional team [1,2].

However, in the last decades, a growing number of scientific publications have been demonstrating that several patients suffer damages caused by health care assistance failures. These damages can increase length of stay in hospitals, permanent sequelae and even death of the patient [1,2].

Thus, providing a safety culture is the first and undoubtedly more important characteristic that patient care should have, since it is the basis for other measures, and is everything that must be done to avoid patients suffering unnecessary damage caused by the assistance of those who should only assist them at this stage of life.

The subject was intensified in the late 1990s in the United States of America (USA), then reached other countries, from the publication of the report "To Err is Human": Building a Safer Health System of the US Institute of Medicine, showing that an estimated of 98,000 people died in a year due to incidents in hospitals. This was more prevalent than dying from vehicle accidents, breast cancer or Acquired Immunodeficiency Syndrome (AIDS) [1,2].

Therefore, the number of deaths due to adverse health events is alarming. It is estimated that approximately 400,000 patients die annually from avoidable adverse events, and between two and four million events have serious consequences for the patient's health. When compared to adults, hospitalized children have increased their likelihood of being harmed three times [3,4].

The adverse event in health services, among the diverse definitions in the world, can be identified as an injury or unintentional or undesirable damage caused to the patient by the care intervention, rather than by the underlying disease [5]. Those that most commonly impact on patient and family life are: health care-associated infections (HAIs), medication events, missed dose or route of administration, serious adverse drug reactions, misdiagnoses, miscommunication among professionals and events related to professional experience [6].

It is necessary that the safe attendance becomes routine in the health services. Therefore, a patient's safety culture is considered an important structural component of the services that favors the implementation of safe practices and the reduction of safety incidents, and is marked by open communication, teamwork, recognition of mutual dependence and the primacy of safety as a priority at all levels of the organization [7].

The existence of a safety culture improves the organizational climate and day-to-day health services, which reduces the chances of exhaustion and illness of the health professional. There are associations between Burnout Syndrome and lower perceptions of safety culture [8] and greater risks in patient safety [9].

The compromise of patient safety derives from manifestations of Burnout, a syndrome characterized by the combination of emotional exhaustion, depersonalization and a reduced sense of personal fulfillment [10]. Emotional exhaustion leads to a feeling of physical and mental exhaustion, depersonalization results in insensitive and distant attitudes of the patient, and in the reduction of personal fulfillment, the healthcare professional feels incompetent and incapable of performing his or her functions [11].

Due to the high prevalence of Burnout in health care workers [12], increasing numbers of adverse events (AEs) especially reaching pediatric patients and the urgent need to measure levels of safety culture in the hospital, a question comes to the table: Does the safety culture of the patient in pediatric hospitals have a relation with the development of Burnout Syndrome?

Therefore, this study aims to analyze the relationship between patient safety culture and burnout in pediatric hospitals.

## Method

The study respected the ethical and legal aspects of research involving human beings, obtaining an approving opinion by the Ethics in Research Committee (CEP) of the Faculty of Juazeiro do Norte (FJN), Brazil, under n° 1,935,510.

This is a cross-sectional study with a quantitative approach performed with healthcare professionals working in pediatric hospitals located in the Metropolitan Region of Cariri, Northeastern Brazil.

The study population comprised professionals from three hospital units that provide assistance in pediatrics linked to the Unified Health System (SUS). Physicians, nurses, physiotherapists, technicians and nursing assistants were candidates for research because they provide direct care to pediatric patients.

Hospitals 1, 2 and 3 have 110, 46 and 110 professionals eligible for research, respectively. Only those with a minimum of six months of service experience were included in the study, considering that this period is sufficient and necessary to understand the hospital routine in relation to the patient's safety culture in the institutions. The worker´s cooperative were excluded, due to discontinuance of shifts in services.

Data collection took place between August and November 2017, through visits to the hospitals, in different shifts (morning, afternoon and evening), during four weeks for each institution.

For the evaluation of the Patient Safety Culture, the version, translated and validated for Brazil, of the Hospital Survey on Patient Safety Culture (HSOPSC), prepared by the Agency for Healthcare Research and Quality (AHRQ) was applied. It is an instrument that covers the 12 dimensions of the patient's safety culture, at work unit and hospital levels, which makes it possible to identify the positive aspects and areas that need improvement [13].

The instrument contains 50 items in total; 44 are related to specific safety culture issues and 6 items are related to personal information. Of the 12 dimensions, 3 are related to the hospital, 7 related to the ward, and two outcome variables. Most items are answered on a 5-point likert scale [14].

For HSPOSC analysis, after the inversion of the reversed items, each dimension was evaluated according to the percentage of positive responses, and the percentage obtained through the combination of the two highest response categories (scores 4 and 5); of each dimension the two lowest categories indicate negative results regarding culture (scores 1 and 2); and the middle category shows neutrality (score 3) [14].

Percentages greater than 75 of positive responses represent "strong patient safety areas," results between 50–75% answered positively were considered "moderate areas", and percentages below 50% demonstrated "fragile areas" [14].

To evaluate the occurrence of Burnout, the Maslach Burnout Inventory (MBI), developed by Christina Maslach and Susan Jackson in 1978, was chosen. It is the most widely used instrument to evaluate burnout regardless of the occupational characteristics of the sample and its origin [15,16,17].

This instrument evaluates the three dimensions or subscales of the syndrome: emotional exhaustion (EE), depersonalization (DP) and low professional achievement (LPA) [18].

Initially the inventory had 47 items, however the current version contains only 22, distributed in: EE with nine questions (1, 2, 3, 6, 8, 13, 14, 16 and 20), DP with five questions (5, 10, 11, 15 and 22) and LPA with eight questions (4, 7, 9, 12, 17, 18, 19 and 21). For each item a likert scale with a score of 1 to 5 was used, one for "never"; two for "a few times a year"; three for "a few times a month"; four for "a few times a week"; and five for "daily" [19].

Each one of the burnout criteria is measured separately, which results in three scores for each participant. These scores, by subscale, were analyzed using the following parameters: EE (<19—Low, 19 to 26—Moderate, ≥27—High); DE (<6—Low, 6 to 9—Moderate, ≥10—High) and BRP (≥40—Low, 34 to 39—Moderate, ≥33—High). The syndrome was considered present, when the participant showed high emotional exhaustion or depersonalization, or low professional achievement [20].

For the organization of the data collected, the two questionnaires were transcribed into a database using Microsoft Excel 2018 software. Then, after collecting the values of the scores in the data processing and analysis, the statistic used was the statistical software Statistical Package for Social Science (SPSS) version 22.0.

Descriptive statistics were used, with absolute frequency, percentage values and Chi-Square test, adopting the significance level of p ≤ 0.05 for a 95% confidence interval.

## Results

The sample consisted of 148 health professionals who worked directly in the pediatric hospital, most of them females (86.5%), with a mean age of 34 years, and the majority from the nursing area, especially technicians (57.4%) followed by physicians, residents or in training (13.5%), with an average of 8 years of experience (Table 1).

**Table 1:** Characterization of the sample. Ceará, Brazil, 2018.

Variable	N	%
**Sex**		
Male	20	13,5
Female	128	86,5
**Age**		
Mínimum	19	-
Maximun	60	-
Average	34,5	-
**Professional Background**		
General Practitioner	11	7,4
Resident Doctor / Doctors in training	9	6,1
Nurse	31	20,9
Nurse Technician	85	57,4
Nursing Assistant	1	0,7
Physiotherapist	8	5,4
Clinical Preceptor	1	0,7
Missing data	2	1,4
**Period of professional experience**		
Minimun	0	-
Maximun	35	-
Average	8,69	-

Most of the professionals work in the pediatric clinic (63.5%), between one and five years (52.7%) in their respective wards (40.5%) in a range of 20 to 59 hours weekly (86.5%), as shown in Table 2.

**Table 2:** Characterization of the sample in relation to the ward and working time period. Ceará, Brazil, 2018.

Variable	N	%
Pediatric Clinic	94	63,5
Pediatric Emergency	24	16,2
Pediatric ICU	30	20,3
**Period of time working in the hospital (years)**		
Less than a year	20	13,5
1 to 5	78	52,7
6 to 10	25	16,9
11 to 15	8	5,4
16 to 20	10	6,8
More than 21	5	3,4
Missing data	2	1,4
**Workload (hours/week)**		
Less than 20	5	3,4
20 a 39	74	50,0
40 a 59	54	36,5
60 a 79	9	6,1
80 a 99	3	2,0
100 or more	1	,7
Missing data	2	1,4
**Period time working in the current Ward(years)**		
Less than a year	26	17,6
1 to 5	60	40,5
6 to 10	30	20,3
11 to 15	12	8,1
16 to 20	8	5,4
21 or more	10	6,8
Missing data	2	1,4

**Fig 1:**
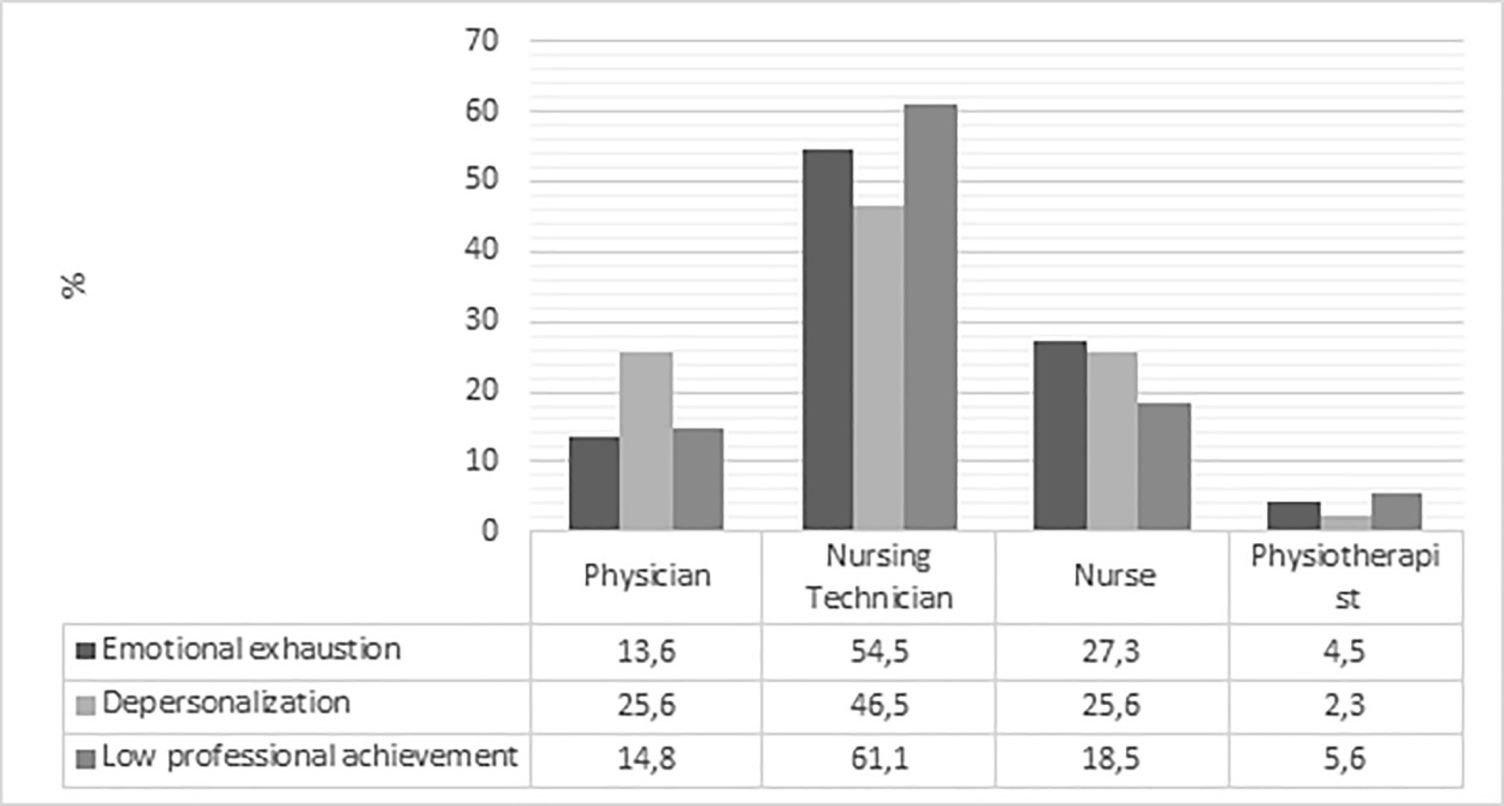
Burnout dimensions. Ceará, Brazil, 2018.

Among the dimensions of burnout presented as moderate to high are highlighted the depersonalization and the low professional achievement, where more than half of the employees showed these two characteristics (Fig 1).

It was verified the presence of burnout in professionals based on their three dimensions (depersonalization, emotional exhaustion and low professional achievement). It was identified that 44, 6% of the profesionals have the disease, identified with “high” score. (Table 3).

**Table 3:** Presence of burnout in professionals. Ceará, Brazil, 2018.

Variable	N	%
**Burnout Clasification** **(1 dimension)**		
No Burnout	82	55,4
Burnout	66	44,6

As showed in Table 4 among the 12 dimensions of patient safety studied, nine were considered fragile, two strong and one moderate, four of them were considered by the professionals as weakest, presenting the highest percentages as follows: D4-Openness of communication (70.3%), D7- professional training (73.5%), D12-Frequency of events notified (69.6%) and D6-Non-punitive response to error (89.9%).

**Table 4:** Patient safety dimensions. Ceará, Brazil, 2018.

	Safety Patients Areas					
Dimensions	Weak Areas		Moderate Areas		Strong Áreas	
	n	%	n	%	n	%
D1-Expectations and promotion of patient safety iniciatives by supervisors and managers.	79	53,4	0	0,0	69	46,6
D2- Organizational learning and improvement.	54	36,5	62	41,9	32	21,6
D3- Team Work within the service units.	58	39,2	0	0,0	90	60,8
D4- Openness in Communication	104	70,3	34	23,0	10	6,8
D5- Feedback and communication about errors.	94	63,5	40	27,0	14	9,5
D6- Non punitive response to error	133	89,9	14	9,5	1	0,7
D7- Professional Training	108	73,5	0	0,0	39	26,5
D8- Support the patient safety culture.	74	50,0	41	27,7	33	22,3
D9- Team work and cooperation among the different service units.	79	53,4	0	0,0	69	46,6
D10- Duty / shift transfers and internal transfers	70,	43,3	0	0,0	78	52,7
D11- General perception of Patient Safety.	82	55,4	49	33,1	17	11,5
D12-Frequency of event notification.	103	69,6	0	0,0	45	30,4

All dimensions of safety of the patient studied were correlated with some dimensions of burnout, where it was observed that the dimension D9-Teamwork in the ward was the most significant because it has an association with the three dimensions of the syndrome (EE: D2-Organizational learning and mutual improvement, D4-Openness in communication, D10- Duty/ shift transfers and D11-General perception of patient safety, presented a correlation with two dimensions of burnout, and the others, with only one (Table 5).

**Table 5:** Correlation between dimensions of patient safety culture and burnout. Ceará, Brazil, 2018.

	Dimensions of Burnout (p <0,05)		
Dimensions of Patient Safety	Emotional Exhaustion	Despersonalization	Low Professional Achievements
D1-Expectations and promotion of patient safety iniciatives by supervisors and managers.	0,405	0,292	**0,024**
D2- Organizational learning and team improvement	0,158	**0,018**	**0,011**
D3-Team work within the service units	**0,010**	0,188	0,115
D4- Openness in Communication	**0,003**	0,475	**0,002**
D5- Feedback and Communication about errors	0,064	0,304	**0,010**
D6- Non punitive response to error	0,788	0,352	**0,026**
D7- Employee training	**0,042**	0,662	0,082
D8- Patient safety culture support	**0,043**	0,062	0,558
D9-Team work and cooperation among the different service units.	**0,007**	**0,049**	**0,039**
D10- Duty / shift transfers and internal transfers	0,061	**0,028**	**0,017**
D11-General perception of patient safety	0,571	**0,009**	**0,031**
D12-Frequency of event notification.	0,116	**0,008**	0,077

## Discussion

Like previous studies with healthcare professionals, the majority is concentrated in female individuals [21,22], as well as age, having an average within the young adult period, as in the study by Sanchez, Tudela, and Seller [23], which presents the average of the interviewed professionals of 44 years, while in the present study the age average is 34.4 years.

In the case of the burnout dimensions proposed by the Maslach Burnout Inventory, the study corroborates the results of Faivre et al. [24] with resident physicians who showed burnout with the majority of the participants classified in the dimensions of depersonalization and low professional achievement.

In public hospitals in France, high emotional exhaustion was observed in 19% of intensivists, high depersonalization in 37% and low professional achievement in 39% [25]. A similar result was also described by Tironi et al., [26] for depersonalization (26.1%) of physicians who worked in intensive care units in five Brazilian capitals.The research also identified the pediatric intensivits, showing similar results in this group, which presented high levels of emotional [26].

Among nurses working in pediatric units, a systematic review with meta-analysis identified moderate to high numbers of emotional exhaustion (31%), depersonalization (21%) and low professional achievement (39%) [27] showing different results to those found in the present study.

The most severely affected dimension with high rates of 29.1% was depersonalization, a condition that leads to negative reactions, cynicism and distancing of patients. Among nurses working in a large hospital in the South of Brazil, the highest subscale was also the depersonalization (21.9%) [28]. Some other studies show divergent results [25,26,29].

The presence of high levels of depersonalization as one of the dimensions of burnout can be associated with the exhausting work process, evidenced directly in the psychological aspect of the professional, which in many situations can generate estrangement and indifference in front of the patient's pain [30]. Other factors may also contribute to a state of depersonalization, such as: difficulty in dealing with the suffering of the other and poor skills to overcome the fatigue by personal or professional situations [31,32].

In a study carried out with pediatric residents, with an average work period of 78 hours per week, it was identified that the level of depersonalization increased in the residents as the number of shifts increased [33].

It is possible to classify an individual with burnout syndrome in different ways. Some authors defend the classification when considering the three dimensions with indices classified as high; or only one of the three [20]; or two of these, being depersonalization and / or emotional exhaustion [34].

In the study, the classification of Grunfeld et al. [20] was generally considered, evidencing that 44.6% of the sample showed the burnout syndrome. Following the study by Cavalcanti et al. [34], it was identified that 29.1% presented depersonalization and 14.9% emotional exhaustion, classifying an average of 44% of those interviewed with evidence of burnout syndrome.

In Brazil, in a sample of intensive care physicians working in pediatrics, it was found a prevalence of 50% of the syndrome [29]. In the United States, among 253 pediatricians studied, 49% had at least one of the criteria for exhaustion, and 21% reported severe burnout [35].

Organizational factors, particularly workload and autonomy, are commonly reported as major risk factors in the literature on burnout [36]. Therefore, although an association between burnout and workload was not tested, it is presumed that this interference may be associated with this factor, considering that approximately 46% of the participants stated that they worked over 40 hours / week.

It has been shown that a high number of shifts implies exhaustion and low job satisfaction [33,37]. The influence of organizational issues on the development of burnout has been tested worldwide, and there are associations between the syndrome and performance in sectors that attend traumatic situations, long and irregular work shifts [38], influence of sectors such as [39] and, more recently, the relationship between the disease and the patient's safety culture has been described [8].

The study presents a different result from what is usually found in other studies evaluating burnout in health professionals, since it evidences higher depersonalization than emotional exhaustion. This fact can be attributed to the wards in which the study was performed, where all belonged to pediatric care [40,41].

There is no evidence yet that is directly associated with this hypothesis, however, some research shows that at the individual level, personality, emotions, and professional charac- teristics (high motivation and investment in work) can contribute to this fact [40,41].

The association with the pediatric public in turn occurs through a greater sense of incapacity to face the suffering of the child, and also of the occurrence of death in this, when compared to the other age groups, since the professionals believe that the death in childhood goes of direction contrary to the natural order, contributing to depersonalization. In addition, it is evident that contact with family members is one of the main factors contributing to such a condition to be awoke in the professional [42–44].

Several studies have shown an association between burnout and patient safety. Regarding patient safety, it was used the Hospital survey on Patient Safety Culture (HSOPSC), showing that of the 12 dimensions of the instrument, nine were considered fragile, especially the dimension "non-punitive response to error".

Sorskar et al., [45] when applying HSOPSC in an emergency medical service, identified that the most fragile dimension was focused on the "hospital management support for patient safety" process, however, the dimension " non punitive response to error " showed a statistical correlation with the level of patient safety, proving to be an important factor to promote safe patient care.

In three pediatric hospitals in Santa Catarina, Brazil, a study that used the HSOPSC to analyze the safety culture of the patient in the nursing team's view, indicated that 61% of the participants presented negative responses to "non-punitive response to error" [46]. This dimension also obtained the lowest score of positive responses (26.8%) in a study conducted in a capital of the Middle East [47].

The error treated in a merely punitive way discourages the professional to make the notifications of the adverse events occurred, which prevents the analysis of the factors that contributed to the occurrence of the error, and thus, the opportunity for learning and improvement is lost. In this sense, a survey of 70 nurses from Intensive Care Units of São Paulo, 52 (74%) reported that punishment occurs most often when some error is reported [48].

The "professional improvement " obtained 73.5% of negative responses, this dimension addresses issues related to the human resources required, and reveals a reality in which there are insufficient professionals, which leads that a person has to execute several tasks simultaneously.

In Brazil, frequently, there are precarious working conditions, limited resources, overcrowding and long waiting times in emergency services of public hospitals, this causes a greater predisposition to incidents and failures in patient care [49]. There are associations between the workload and the occurrence of higher numbers of adverse events, such as infections, pressure injuries and errors related to medication administration [50].

Therefore, fragility in this dimension constitutes a real risk to the patient's safety, thus, as a factor generating professional dissatisfaction. In this sense, some studies observe that high levels of job satisfaction are correlated with low burnout in pediatric nurses [51].

A negative correlation was also found between emotional exhaustion and job satisfaction [52].

When analyzing the relationship between burnout and patient safety, it was possible to identify statistical correlation of the 12 dimensions of HSOPSC with at least one of the three subscales of the syndrome.

The dimension "teamwork among the different service units”, perceived as fragile (53.4%) by most participants, was associated with all burnout subscales. This factor may show that the low capacity of articulation among the different hospital wards to cooperate for a quality assistance and the development of burnout, lead the professional to have a greater tendency to the development of the burnout syndrome [53–55].

Given the vulnerability of the child, especially those hospitalized, the professionals who assist them directly demand the articulation and cooperation of other hospital wards and even other institutions, so that comprehensive and qualified assistance is offered.

In this way, when the professional perceives failures in this cooperation, he may feel exhausted, unable to take all the necessary care, which culminates in the feeling of incompetence. From this, emotional exhaustion can arise, which may lead to depersonalization and, finally, low professional achievement [53–55].

This fact confirms the hypothesis that the hospital organization directly influences the psychological behavior of the professionals (burnout), as well as patient safety, considering that the collaborative work among units is one of the key factors of management organization, as shown in the study by Liu et al., [55] performed with nurses at a Chinese hospital.

The influence of teamwork on burnout is seen in other studies. Disrespectful interaction among co-workers was described as a significant factor for emotional exhaustion in a study of 197 Malaysian public hospital doctors [56]. In Hungary, role conflicts among the team members was associated with emotional exhaustion, from a research conducted with 201 health professionals of two hospitals [57].

The dimensions "organizational learning", "openness to communication", "shift transfers" and "general perception of patient safety", were related to two subscales of burnout, showing the relationship between organizational climate and disease development, and demonstrating also the hypothesis described previously.

This influence is reinforced by a study carried out in 44 neonatal intensive care units, in which those units with a higher prevalence of burnout presented a lower teamwork climate, safety climate, job satisfaction, management perceptions and working conditions [8].

In Baton Rouge, USA, the study points out the importance of expanding the discussion on patient safety in health services, evidencing that when performing interventions / training with US residents regarding communication and patient safety, professionals reported better communication and an increase in the number of safety events reported at the end of the intervention [58].

Thus, it is noticeable that the management organization of a hospital service directly influences patient safety. Thus, some authors have already defended the organization of services aimed at patient safety that are effective in their management, promoting psychological well-being for professionals and safe care.

An example of this is the Leadership Walk Rounds (WR) program, which has been widely adopted, especially in the USA, to standardize visits to health care, work observation and request for ideas to improve the quality of care. A study conducted in 31 US hospitals found that the use of WR with feedback to professionals was associated with better safety culture assessments, greater involvement in the workforce and less fatigue [59].

Still, the authors state that feeling like you have control over the quality of care through WR may reduce perceptions of burnout, for example, that you are overworked or frustrated by your work [59]. In view of this, it can be seen that the organizational management deficit can be a great contributing factor for the development of the burnout syndrome.

From the point of view of Public Health, understanding the relationship between safety culture and the psychological health of healthcare professionals can provide leaders with an opportunity to intervene synergistically in the fields of occupational health and patient safety as they are interconnected. Therefore, strategies to promote a patient's safety culture are recommended, which can reduce the risks for the development of Burnout and an effective improvement in the quality of care [60].

The research had limitations mainly for its data collection, due to the resistance of the participants, because they claimed the exhaustive work routine to not return the questionnaires. Also, although a large number of burnout studies have been observed, the percentage of those that associate with patient safety is minimal, leading, therefore, to the need to perform this type of study with other profile of employees and services.

## Conclusion

The study evidenced the influence of all patient safety domains for the development of burnout syndrome in pediatric professionals. These professionals, in turn, have awakened in most cases, depersonalization, unlike other surveys, which show emotional exhaustion as the most frequent subscale.

Also, it was identified that the organizational climate is the main determinant of burnout, especially in what refers to "teamwork among wards", showing that an organized management can promote psychological well-being of the professionals and safe assistance to the patients.
